# Tetra­ammonium μ-ethyl­enedi­amine­tetra­acetato-1κ^3^ *O*,*N*,*O*′:2κ^3^ *O*′′,*N*′,*O*′′′-bis­[trioxidotungstate(VI)] tetra­hydrate

**DOI:** 10.1107/S2414314621009822

**Published:** 2021-09-28

**Authors:** Lamine Yaffa, Sérigne Fallou Pouye, Daouda Ndoye, Waly Diallo, Mayoro Diop, Mamadou Sidibe, Cheikh Abdoul Khadir Diop

**Affiliations:** a Laboratoire de Chimie Minérale et Analytique (LACHIMIA), Département de Chimie, Faculté des Sciences et Techniques, Université Cheikh Anta Diop, Dakar, Senegal; University Koblenz-Landau, Germany

**Keywords:** crystal structure, ethyl­ene­di­amine­tetra­acetate, tungstic acid, binuclear complex

## Abstract

The title compound, (NH_4_)_4_[W_2_(C_10_H_12_N_2_O_8_)O_6_]·4H_2_O, was obtained from a mixture of tungstic acid, ammonia and ethyl­enedi­amine­tetra­acetic acid (H_4_edta) in a 2:4:1 ratio. The anion of the complex contains two WO_3_ units and one bridging edta^4−^ ligand. Each central metal atom is tridentately coordinated by nitro­gen and two carboxyl­ate groups of the edta^4−^ ligand, together with the three oxido ligands, producing a distorted octa­hedral coordination environment around each tungsten atom. The center of the carbon–carbon bond of the ethyl­ene bridge represents a crystallographic inversion center. The crystal structure consists of a three-dimensional supra­molecular framework built up by the dinuclear cations, the ammonium counter-ions and the solvent water mol­ecules *via* hydrogen bonds of the N—H⋯O and O—H⋯O type.

## Abstract 2

The title compound, (NH_4_)_4_[W_2_(C_10_H_12_N_2_O_8_)O_6_]·4H_2_O, is a binuclear complex of tungsten with the edta^4−^ ligand bridging two WO_3_ units, leading to a distorted octa­hedral coordination environment for the tungsten atoms. The supra­molecular crystal structure is built up by the anion, four ammonium cations and four solvent water mol­ecules and is established *via* hydrogen bonds of the N—H⋯O and O—H⋯O type.

## Graphical abstract

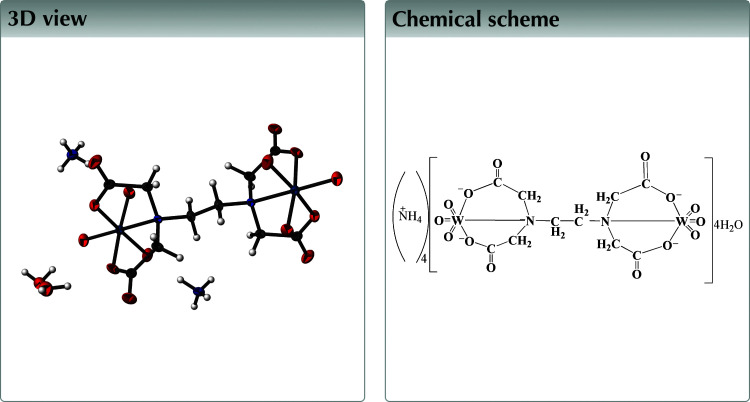

## Structure description

Research on inorganic–organic framework materials is one of the fastest growing areas in materials chemistry because of their unique hybrid nature, which enables the combination of properties from both inorganic and organic materials (Cheetham & Rao, 2007 ▸). As organic ligands, polycarboxyl­ates are multidentate chelating agents that are widespread in nature and industry because of their ability to coordinate with various transition metals in different ratios (Nicolau & Guy, 1995 ▸; Langer, 2000 ▸).

As a part of this field, molybdenum polycarboxyl­ate complexes have thus been thoroughly investigated over the past three decades (Lee & Holm, 2004 ▸). Some well-characterized mono-, bi- and polynuclear molybdenum and tungsten complexes have been reported, for example Mo_2_(O_2_CCH_2_OH)_4_, *M*_2_[MoO_3_(C_2_O_4_)] (*M* = Na, K, Rb, Cs), Na_2_[*M*O_2_(C_6_H_6_O_7_)_2_]·3H_2_O (*M* = Mo, W) (Cotton *et al.*, 2002 ▸; Cindríc *et al.*, 2000 ▸; Zhou *et al.*, 1999 ▸). Structural analyses of W^VI^–edta complexes are rare in the literature. Together with the structure of Na_2_K_2_[Mo_2_O_6_(edta)]·10H_2_O, the structure of Na_4_[W_2_O_6_(edta)]·8H_2_O has been published (Lin *et al.*, 2006 ▸).

Nevertheless, tungsten has been reported to incorporate into several enzymes (Johnson *et al.*, 1996 ▸). In fact, tungsten could be a useful probe for the active site of molybdenum enzymes. As a consequence, more effort has been put into tungsten chemistry by inorganic and bioinorganic chemists (Bagno & Bonchio, 2000 ▸; Enemark *et al.*, 2004 ▸; Sung & Holm, 2001 ▸; Zhou *et al.*, 2004 ▸).

In this study, the reaction of H_4_edta with tungstic acid has been investigated and a new binuclear 2:1 W–edta complex, (NH_4_)_4_[W_2_(C_10_H_12_N_2_O_8_)O_6_]·4H_2_O, was isolated and structurally characterized.

**Figure 1:**
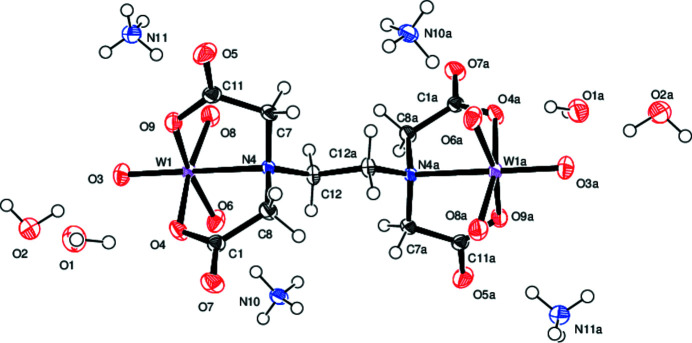
Mol­ecular structure of the title compound. Displacement ellipsoids are drawn at the 50% probability level.

As shown in Fig. 1 ▸, the dinuclear anion of the title compound shows one edta^4−^ ligand bonded to two tungstate WO_3_ units. Each W atom is six-coordinate in a distorted octa­hedral environment built up by the tridentate facial coordination of one N and two O atoms of the edta^4−^ ligand as well as by three oxido ligands. The edta^4−^ ligand itself therefore acts as a bridge between the two WO_3_ units, with the central carbon–carbon bond also representing a crystallographic center of inversion. The anion is accompanied by four ammonium cations and four solvent water mol­ecules.

The three terminal oxido ligands bonded to the metal, *i.e.* W=Ot (Ot = O3, O6, O8) show bond lengths in a range 1.753 (2) to 1.759 (2) Å. The resulting Ot—W—Ot bond angles [105.05 (9), 105.14 (9), 103.12 (10)°] are considerably larger than 90° expected for a regular octa­hedron. Bond distances of the oxygen atoms of edta^4−^ to W are 2.135 (2) and 2.159 (2) Å, respectively and therefore significantly longer than the W=Ot bonds.

**Figure 2:**
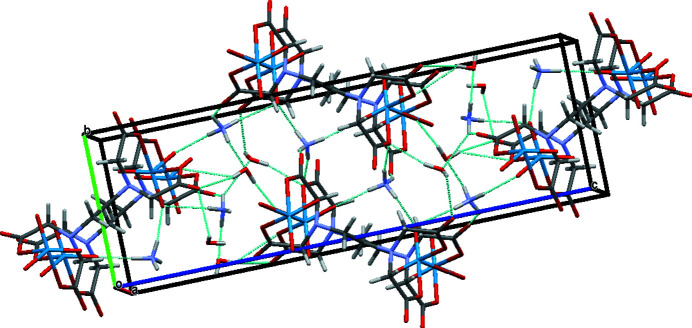
Supra­molecular arrangement of the title compound established by classical hydrogen-bonding inter­actions (dashed lines).

In the crystal structure, the complex anion, ammonium cations and solvent water mol­ecules inter­act through medium–strong classical hydrogen bonds (Table 1 ▸). Two neighboring complexes are connected *via* hydrogen bonds of the N—H⋯Ow—H⋯O, N—H⋯O and Ow—H⋯O types. These inter­actions lead to the supra­molacular structure shown in Fig. 2 ▸.

## Synthesis and crystallization

Tungstic acid (4 mmol, 0.999 g) and ammonia solution (8 mmol, 1.001 g) were mixed in 30 ml of water to solubilize the W^VI^ source. To this mixture was slowly added ethyl­enediammine-tetra­acetic acid (H_4_edta) (2 mmol, 0.584 g) under vigorous stirring. The solution was then stirred for two h at room temperature. The colorless solution thus obtained was left at room temperature for slow evaporation of water. After two weeks, colorless crystals (yield 11.6% based on W) were obtained from the solution.

The FT–infrared spectra of the title compound shows well-resolved absorption bands for the carboxyl­ate of the coord­inating edta^4−^ at 1651 cm^−1^ and 1402 cm^−1^, which are attributed to the anti­symmetric and symmetric stretching vibrations ν(COO–). The bands at 926, 857 and 666 cm^−1^ can be attributed to symmetric and asymmetric W=Ot stretching vibrations (Lin *et al.*, 2006 ▸; Li *et al.*, 2007 ▸). The range of 3500–2800 cm^−1^ shows many bands ascribed to O—H stretching of water mol­ecules, as well as N—H stretching vibrations of ammonium cations (Yaffa *et al.*, 2020 ▸).

## Refinement

Crystal data, data collection and structure refinement details are summarized in Table 2 ▸.

## Supplementary Material

Crystal structure: contains datablock(s) I. DOI: 10.1107/S2414314621009822/im4013sup1.cif

Structure factors: contains datablock(s) I. DOI: 10.1107/S2414314621009822/im4013Isup2.hkl

CCDC reference: 2112632

Additional supporting information:  crystallographic information; 3D view; checkCIF report

## Figures and Tables

**Table 1:** Hydrogen-bond geometry (Å, °)

*D*—H⋯*A*	*D*—H	H⋯*A*	*D*⋯*A*	*D*—H⋯*A*
O2—H2*A*⋯O3	0.87	1.88	2.743 (3)	171
O2—H2*B*⋯O5^i^	0.87	1.90	2.763 (3)	170
O1—H1*A*⋯O7	0.87	2.72	3.468 (3)	145
O1—H1*A*⋯O4	0.87	2.06	2.900 (3)	161
O1—H1*B*⋯O2^ii^	0.87	2.49	3.177 (3)	137
N10—H10*A*⋯O6	0.95 (4)	1.78 (4)	2.727 (3)	175 (3)
N10—H10*B*⋯O4^iii^	0.81 (4)	2.20 (4)	2.996 (3)	168 (3)
N10—H10*C*⋯O7^iv^	0.90 (4)	2.01 (4)	2.876 (3)	160 (3)
N10—H10*D*⋯O8^v^	0.87 (4)	1.87 (4)	2.736 (3)	175 (4)
N11—H11*A*⋯O8	0.87 (5)	2.13 (5)	2.947 (3)	156 (4)
N11—H11*B*⋯O3^vi^	0.84 (5)	2.31 (5)	3.109 (3)	160 (4)
N11—H11*C*⋯O1^vi^	0.83 (4)	2.25 (4)	2.979 (4)	147 (4)
N11—H11*D*⋯O2^vii^	0.92 (4)	1.93 (4)	2.846 (4)	173 (4)

Symmetry codes: (i) 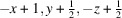 ; (ii) 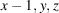 ; (iii) 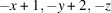 ; (iv) 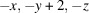 ; (v) 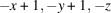 ; (vi) 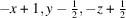 ; (vii) 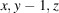 .

**Table 2:** Experimental details

Crystal data	
Chemical formula	(NH_4_)_4_[W_2_(C_10_H_12_N_2_O_8_)O_6_]·4H_2_O
*M*_r_	896.15
Crystal system, space group	Monoclinic, *P*2_1_/*c*
Temperature (K)	150
*a*, *b*, *c* (Å)	6.8017 (5), 7.7194 (5), 23.9807 (19)
β (°)	95.345 (3)
*V* (Å^3^)	1253.63 (16)
*Z*	2
Radiation type	Mo *K*α
μ (mm^−1^)	9.26
Crystal size (mm)	0.18 × 0.18 × 0.14

Data collection	
Diffractometer	Bruker APEXII CCD
Absorption correction	Multi-scan (*SADABS*; Bruker, 2016 ▸)
*T*_min_, *T*_max_	0.444, 0.746
No. of measured, independent and observed [*I* > 2σ(*I*)] reflections	55419, 2890, 2703
*R*_int_	0.053
(sin θ/λ)_max_ (Å^−1^)	0.652

Refinement	
*R*[*F* ^2^ > 2σ(*F* ^2^)], *wR*(*F* ^2^), *S*	0.014, 0.034, 1.07
No. of reflections	2890
No. of parameters	201
H-atom treatment	H atoms treated by a mixture of independent and constrained refinement
Δρ_max_, Δρ_min_ (e Å^−3^)	0.71, −0.85

Computer programs: *APEX2* and *SAINT* (Bruker, 2016 ▸), *SHELXS* (Sheldrick, 2008 ▸), *SHELXL2014/6* (Sheldrick, 2015 ▸) and *OLEX2* (Dolomanov *et al.*, 2009 ▸).

## full crystallographic data

### Crystal data

**Table:**

(NH_4_)_4_[W_2_(C_10_H_12_N_2_O_8_)O_6_]·4H_2_O	*F*(000) = 860
*M**_r_* = 896.15	*D*_x_ = 2.374 Mg m^−^^3^
Monoclinic, *P*2_1_/*c*	Mo *K*α radiation, λ = 0.71073 Å
*a* = 6.8017 (5) Å	Cell parameters from 9817 reflections
*b* = 7.7194 (5) Å	θ = 2.8–27.6°
*c* = 23.9807 (19) Å	µ = 9.26 mm^−^^1^
β = 95.345 (3)°	*T* = 150 K
*V* = 1253.63 (16) Å^3^	Block, colourless
*Z* = 2	0.18 × 0.18 × 0.14 mm

### Data collection

**Table:**

Bruker APEXII CCD diffractometer	2703 reflections with *I* > 2σ(*I*)
φ and ω scans	*R*_int_ = 0.053
Absorption correction: multi-scan (SADABS; Bruker, 2016)	θ_max_ = 27.6°, θ_min_ = 2.8°
*T*_min_ = 0.444, *T*_max_ = 0.746	*h* = −8→8
55419 measured reflections	*k* = −10→10
2890 independent reflections	*l* = −31→31

### Refinement

**Table:**

Refinement on *F*^2^	Primary atom site location: structure-invariant direct methods
Least-squares matrix: full	Hydrogen site location: mixed
*R*[*F*^2^ > 2σ(*F*^2^)] = 0.014	H atoms treated by a mixture of independent and constrained refinement
*wR*(*F*^2^) = 0.034	*w* = 1/[σ^2^(*F*_o_^2^) + (0.0138*P*)^2^ + 1.4846*P*] where *P* = (*F*_o_^2^ + 2*F*_c_^2^)/3
*S* = 1.07	(Δ/σ)_max_ = 0.002
2890 reflections	Δρ_max_ = 0.71 e Å^−^^3^
201 parameters	Δρ_min_ = −0.85 e Å^−^^3^
0 restraints	

### Special details

**Table:**

Geometry. All esds (except the esd in the dihedral angle between two l.s. planes) are estimated using the full covariance matrix. The cell esds are taken into account individually in the estimation of esds in distances, angles and torsion angles; correlations between esds in cell parameters are only used when they are defined by crystal symmetry. An approximate (isotropic) treatment of cell esds is used for estimating esds involving l.s. planes.
Refinement. All non-hydrogen atoms were refined anisotropically. Hydrogen atoms bonded to carbon and oxygen were placed in idealized positions and refined using a riding model with isotropic displacement parameters calculated as *U*_iso_(H) = 1.2*U*_eq_(C) for ethylene and methylene hydrogen atoms and *U*_iso_(H) = 1.5*U*_eq_(O) for solvent water molecules. Hydrogen atoms of the ammonium cations were located in the difference-Fourier map and refined isotropically.

### Fractional atomic coordinates and isotropic or equivalent isotropic displacement parameters (Å^2^)

**Table:**

	*x*	*y*	*z*	*U*_iso_*/*U*_eq_	
W1	0.40177 (2)	0.69956 (2)	0.10889 (2)	0.01546 (4)	
O6	0.4717 (3)	0.7513 (3)	0.04235 (8)	0.0290 (4)	
O3	0.5793 (3)	0.7989 (2)	0.15614 (9)	0.0270 (4)	
O9	0.2095 (3)	0.6721 (2)	0.17381 (8)	0.0237 (4)	
N4	0.0791 (3)	0.6278 (2)	0.06743 (8)	0.0143 (4)	
O8	0.4519 (3)	0.4764 (2)	0.11461 (8)	0.0276 (4)	
O5	−0.0230 (3)	0.5116 (3)	0.20717 (8)	0.0351 (5)	
C7	−0.0056 (4)	0.5133 (3)	0.10897 (10)	0.0192 (5)	
H7A	0.0366	0.3924	0.1033	0.023*	
H7B	−0.1515	0.5173	0.1031	0.023*	
C11	0.0609 (4)	0.5690 (3)	0.16809 (11)	0.0200 (5)	
C12	0.0980 (4)	0.5353 (3)	0.01373 (11)	0.0190 (5)	
H12A	0.1915	0.4378	0.0208	0.023*	
H12B	0.1546	0.6157	−0.0127	0.023*	
O7	−0.0275 (3)	1.0904 (2)	0.07169 (9)	0.0280 (4)	
O4	0.2366 (3)	0.9388 (2)	0.10315 (9)	0.0253 (4)	
C1	0.0597 (4)	0.9519 (3)	0.07921 (11)	0.0183 (5)	
C8	−0.0402 (4)	0.7886 (3)	0.05795 (12)	0.0219 (5)	
H8A	−0.1639	0.7748	0.0762	0.026*	
H8B	−0.0769	0.8012	0.0172	0.026*	
N10	0.4415 (4)	0.8304 (3)	−0.06894 (11)	0.0225 (5)	
O2	0.7912 (3)	1.0837 (3)	0.19478 (9)	0.0357 (5)	
H2A	0.7148	0.9966	0.1847	0.054*	
H2B	0.8584	1.0492	0.2254	0.054*	
O1	0.2481 (3)	1.1729 (3)	0.19796 (10)	0.0367 (5)	
H1A	0.2243	1.1196	0.1661	0.055*	
H1B	0.1322	1.2036	0.2070	0.055*	
N11	0.5898 (4)	0.3808 (4)	0.23069 (12)	0.0262 (5)	
H10A	0.456 (5)	0.808 (4)	−0.0300 (16)	0.032 (9)*	
H10B	0.521 (5)	0.904 (5)	−0.0757 (14)	0.034 (9)*	
H10C	0.316 (6)	0.864 (5)	−0.0785 (16)	0.045 (10)*	
H10D	0.471 (6)	0.735 (5)	−0.0853 (17)	0.046 (11)*	
H11A	0.515 (6)	0.409 (6)	0.2004 (19)	0.060 (13)*	
H11B	0.517 (7)	0.355 (6)	0.256 (2)	0.069 (14)*	
H11C	0.662 (6)	0.466 (6)	0.2389 (17)	0.054 (12)*	
H11D	0.663 (6)	0.288 (5)	0.2208 (17)	0.047 (11)*	

### Atomic displacement parameters (Å^2^)

**Table:**

	*U*^11^	*U*^22^	*U*^33^	*U*^12^	*U*^13^	*U*^23^
W1	0.01185 (5)	0.01695 (6)	0.01687 (6)	−0.00047 (3)	−0.00242 (4)	0.00027 (4)
O6	0.0201 (10)	0.0457 (11)	0.0211 (10)	−0.0102 (9)	0.0011 (8)	0.0012 (9)
O3	0.0216 (10)	0.0330 (11)	0.0253 (10)	−0.0030 (8)	−0.0044 (8)	−0.0031 (8)
O9	0.0244 (10)	0.0285 (10)	0.0182 (9)	−0.0082 (8)	0.0019 (8)	−0.0016 (7)
N4	0.0136 (9)	0.0125 (9)	0.0161 (10)	−0.0008 (7)	−0.0026 (8)	0.0005 (8)
O8	0.0255 (10)	0.0208 (9)	0.0344 (11)	0.0069 (8)	−0.0087 (8)	−0.0021 (8)
O5	0.0297 (11)	0.0522 (13)	0.0233 (10)	−0.0139 (10)	0.0015 (8)	0.0115 (10)
C7	0.0173 (12)	0.0174 (12)	0.0225 (13)	−0.0051 (9)	−0.0006 (10)	0.0026 (10)
C11	0.0174 (12)	0.0193 (12)	0.0227 (13)	0.0011 (9)	−0.0011 (10)	0.0044 (10)
C12	0.0165 (12)	0.0215 (12)	0.0186 (12)	−0.0022 (9)	−0.0010 (10)	−0.0051 (10)
O7	0.0256 (10)	0.0166 (9)	0.0411 (12)	0.0055 (8)	−0.0012 (8)	0.0011 (8)
O4	0.0190 (9)	0.0153 (9)	0.0396 (12)	−0.0016 (7)	−0.0083 (8)	−0.0005 (8)
C1	0.0169 (12)	0.0176 (12)	0.0202 (13)	0.0004 (9)	0.0013 (9)	0.0015 (9)
C8	0.0177 (12)	0.0142 (12)	0.0320 (15)	0.0019 (9)	−0.0070 (11)	0.0018 (10)
N10	0.0215 (12)	0.0178 (11)	0.0279 (14)	−0.0025 (9)	0.0010 (10)	0.0034 (10)
O2	0.0462 (13)	0.0282 (11)	0.0316 (12)	−0.0025 (10)	−0.0022 (10)	−0.0008 (9)
O1	0.0333 (12)	0.0386 (12)	0.0377 (13)	−0.0046 (10)	0.0013 (10)	−0.0024 (10)
N11	0.0272 (13)	0.0256 (13)	0.0254 (13)	−0.0032 (11)	0.0000 (11)	0.0033 (11)

### Geometric parameters (Å, º)

**Table:**

W1—O6	1.7529 (19)	O7—C1	1.228 (3)
W1—O3	1.7534 (19)	O4—C1	1.288 (3)
W1—O9	2.1350 (19)	C1—C8	1.499 (3)
W1—N4	2.3884 (19)	C8—H8A	0.9900
W1—O8	1.7590 (19)	C8—H8B	0.9900
W1—O4	2.1590 (17)	N10—H10A	0.95 (4)
O9—C11	1.284 (3)	N10—H10B	0.81 (4)
N4—C7	1.487 (3)	N10—H10C	0.90 (4)
N4—C12	1.488 (3)	N10—H10D	0.87 (4)
N4—C8	1.489 (3)	O2—H2A	0.8701
O5—C11	1.225 (3)	O2—H2B	0.8698
C7—H7A	0.9900	O1—H1A	0.8701
C7—H7B	0.9900	O1—H1B	0.8698
C7—C11	1.510 (4)	N11—H11A	0.87 (5)
C12—C12^i^	1.531 (5)	N11—H11B	0.84 (5)
C12—H12A	0.9900	N11—H11C	0.83 (4)
C12—H12B	0.9900	N11—H11D	0.92 (4)
			
O6—W1—O3	105.05 (9)	N4—C12—C12^i^	113.7 (3)
O6—W1—O9	157.36 (9)	N4—C12—H12A	108.8
O6—W1—N4	89.46 (8)	N4—C12—H12B	108.8
O6—W1—O8	103.12 (10)	C12^i^—C12—H12A	108.8
O6—W1—O4	86.03 (9)	C12^i^—C12—H12B	108.8
O3—W1—O9	90.22 (8)	H12A—C12—H12B	107.7
O3—W1—N4	157.08 (8)	C1—O4—W1	123.67 (15)
O3—W1—O8	105.14 (9)	O7—C1—O4	123.5 (2)
O3—W1—O4	89.42 (8)	O7—C1—C8	119.0 (2)
O9—W1—N4	71.28 (7)	O4—C1—C8	117.4 (2)
O9—W1—O4	77.36 (8)	N4—C8—C1	115.2 (2)
O8—W1—O9	88.46 (8)	N4—C8—H8A	108.5
O8—W1—N4	88.23 (8)	N4—C8—H8B	108.5
O8—W1—O4	159.79 (8)	C1—C8—H8A	108.5
O4—W1—N4	73.71 (7)	C1—C8—H8B	108.5
C11—O9—W1	120.89 (17)	H8A—C8—H8B	107.5
C7—N4—W1	104.91 (14)	H10A—N10—H10B	108 (3)
C7—N4—C12	111.36 (19)	H10A—N10—H10C	108 (3)
C7—N4—C8	110.97 (19)	H10A—N10—H10D	106 (3)
C12—N4—W1	108.76 (14)	H10B—N10—H10C	112 (3)
C12—N4—C8	110.94 (19)	H10B—N10—H10D	108 (4)
C8—N4—W1	109.70 (14)	H10C—N10—H10D	113 (4)
N4—C7—H7A	109.4	H2A—O2—H2B	104.5
N4—C7—H7B	109.4	H1A—O1—H1B	104.5
N4—C7—C11	111.03 (19)	H11A—N11—H11B	109 (4)
H7A—C7—H7B	108.0	H11A—N11—H11C	106 (4)
C11—C7—H7A	109.4	H11A—N11—H11D	106 (4)
C11—C7—H7B	109.4	H11B—N11—H11C	113 (4)
O9—C11—C7	116.1 (2)	H11B—N11—H11D	112 (4)
O5—C11—O9	124.1 (3)	H11C—N11—H11D	111 (4)
O5—C11—C7	119.7 (2)		
			
W1—O9—C11—O5	159.8 (2)	C7—N4—C12—C12^i^	58.5 (3)
W1—O9—C11—C7	−18.0 (3)	C7—N4—C8—C1	111.6 (2)
W1—N4—C7—C11	35.7 (2)	C12—N4—C7—C11	153.2 (2)
W1—N4—C12—C12^i^	173.6 (2)	C12—N4—C8—C1	−124.0 (2)
W1—N4—C8—C1	−3.8 (3)	O7—C1—C8—N4	178.2 (2)
W1—O4—C1—O7	−173.4 (2)	O4—C1—C8—N4	0.2 (4)
W1—O4—C1—C8	4.4 (3)	C8—N4—C7—C11	−82.7 (2)
N4—C7—C11—O9	−16.0 (3)	C8—N4—C12—C12^i^	−65.6 (3)
N4—C7—C11—O5	166.0 (2)		

Symmetry code: (i) −*x*, −*y*+1, −*z*.

### Hydrogen-bond geometry (Å, º)

**Table:**

*D*—H···*A*	*D*—H	H···*A*	*D*···*A*	*D*—H···*A*
C7—H7*A*···O7^ii^	0.99	2.48	3.384 (3)	152
C12—H12*B*···O7^iii^	0.99	2.77	3.548 (3)	136
C8—H8*A*···O6^iv^	0.99	2.54	3.319 (3)	135
C8—H8*B*···O7^iii^	0.99	2.46	3.319 (4)	145
O2—H2*A*···O3	0.87	1.88	2.743 (3)	171
O2—H2*B*···O5^v^	0.87	1.90	2.763 (3)	170
O1—H1*A*···O7	0.87	2.72	3.468 (3)	145
O1—H1*A*···O4	0.87	2.06	2.900 (3)	161
O1—H1*B*···O2^iv^	0.87	2.49	3.177 (3)	137
N10—H10*A*···O6	0.95 (4)	1.78 (4)	2.727 (3)	175 (3)
N10—H10*B*···O4^vi^	0.81 (4)	2.20 (4)	2.996 (3)	168 (3)
N10—H10*C*···O7^iii^	0.90 (4)	2.01 (4)	2.876 (3)	160 (3)
N10—H10*D*···O8^vii^	0.87 (4)	1.87 (4)	2.736 (3)	175 (4)
N11—H11*A*···O8	0.87 (5)	2.13 (5)	2.947 (3)	156 (4)
N11—H11*B*···O3^viii^	0.84 (5)	2.31 (5)	3.109 (3)	160 (4)
N11—H11*C*···O1^viii^	0.83 (4)	2.25 (4)	2.979 (4)	147 (4)
N11—H11*D*···O2^ii^	0.92 (4)	1.93 (4)	2.846 (4)	173 (4)

Symmetry codes: (ii) *x*, *y*−1, *z*; (iii) −*x*, −*y*+2, −*z*; (iv) *x*−1, *y*, *z*; (v) −*x*+1, *y*+1/2, −*z*+1/2; (vi) −*x*+1, −*y*+2, −*z*; (vii) −*x*+1, −*y*+1, −*z*; (viii) −*x*+1, *y*−1/2, −*z*+1/2.
